# Breast-conserving surgery versus mastectomy for treatment of breast cancer after neoadjuvant chemotherapy

**DOI:** 10.3389/fonc.2023.1178230

**Published:** 2023-07-11

**Authors:** Yu-Chun Song, Zhou Huang, Hui Fang, Yu Tang, Hao Jing, Yong-Wen Song, Jing Jin, Yue-Ping Liu, Bo Chen, Yuan Tang, Shu-Nan Qi, Ning-Ning Lu, Ning Li, Ye-Xiong Li, Shu-Lian Wang

**Affiliations:** ^1^ Department of Radiation Oncology, National Cancer Center/National Clinical Research Center for Cancer/Cancer Hospital, Chinese Academy of Medical Sciences and Peking Union Medical College, Beijing, China; ^2^ Key Laboratory of Carcinogenesis and Translational Research (Ministry of Education/Beijing), Department of Radiation Oncology, Peking University Cancer Hospital and Institute, Beijing, China; ^3^ Department of Radiation Oncology, National Cancer Center/National Clinical Research Center for Cancer/Cancer Hospital &Shenzhen Hospital, Chinese Academy of Medical Sciences and Peking Union Medical College, Shenzhen, China

**Keywords:** breast cancer, neoadjuvant chemotherapy, breast-conserving surgery, mastectomy, oncological outcomes

## Abstract

**Background:**

To compare recurrence and survival outcomes between breast-conserving surgery (BCS) and mastectomy after neoadjuvant chemotherapy (NACT).

**Methods:**

The data of 730 patients who underwent NACT between 2000 and 2014 were retrospectively reviewed. A total of 104 (14.2%) patients received BCS and 626 (85.8%) received mastectomy. Locoregional recurrence (LRR), distant metastases (DM), disease-free survival (DFS), breast cancer–specific survival (BCSS), and overall survival (OS) were analyzed using the Kaplan–Meier method. The impact of BCS versus mastectomy on outcomes was assessed by multivariate Cox models. Inverse probability of treatment weighting (IPTW) was used to balance covariates between the two groups.

**Results:**

The median follow-up of BCS and mastectomy groups were 86.5 and 87.4 months, respectively. There were significant differences in distribution of most baseline characteristics between two groups. Compared with those who underwent mastectomy, the patients with BCS had similar 5-year LRR, DM, and DFS rates, but had significantly higher 5-year BCSS (98.9% vs. 90.4%, P = 0.005) and OS (98.9% vs. 90.1%, P = 0.003) rates. Multivariate analysis also showed that BCS significantly improved BCSS (HR = 0.27, 95% CI: 0.08-0.85, P = 0.025) and OS (HR = 0.25, 95% CI: 0.08-0.79, P = 0.018). After IPTW adjustment, the LRR, DM, DFS, BCSS and OS between two groups had no significant differences.

**Conclusions:**

The recurrence and survival outcomes are comparable with BCS and mastectomy. Thus, BCS is a safe treatment option for selected breast cancer patients after NACT.

## Introduction

Several prospective randomized clinical trials have demonstrated that breast-conserving surgery (BCS) plus radiotherapy can provide long-term survival outcomes comparable to that with mastectomy (1–3) and better cosmetic outcomes and quality of life (4–6) in early-stage breast cancer (BC). Some recent large population-based studies have even shown better survival rates with BCS (7–10).

Traditionally, neoadjuvant chemotherapy (NACT) was a standard treatment of unresectable locally advanced breast cancer (LABC) to convert these patients to candidates for surgery. Two prospective randomized trials, the NSABP B18 and EORTC 10902, compared NACT and adjuvant chemotherapy in operable BC, and reported similar survival outcomes between two groups (11, 12). Moreover, these two trials both also found that NACT increased the rate of BCS by reducing the size of primary tumor. Based on the findings, NACT has been more widely accepted for operable BC and to facilitate BCS for the patients with large tumors who are initially considered for mastectomy (13, 14). In addition, it has potential to reduce resected volumes for the patients who are already candidates for BCS and achieve better cosmetic outcomes (15). Meanwhile, NACT can determine the chemo-sensitivity and reduce micrometastasis.

However, it is a main concern that whether BCS after NACT would increase the rate of ipsilateral breast tumor recurrence. A meta-analysis including ten studies (from 1983 to 2002) by the Early Breast Cancer Trialists’ Collaborative Group (EBCTCG) found that 69% of patients achieved complete or partial clinical response after NACT and that the frequency of BCS increased from 49% to 65% after NACT. Nevertheless, the 15-year local recurrence rate was higher with NACT than with adjuvant chemotherapy (21.4% vs. 15.9%) (16). Other studies also showed unexpectedly high local recurrence rates for patients who received BCS after NACT (17, 18). One of the most important challenges for surgeons is to determine the original tumor location and excision extent to obtain tumor-free margins and achieve good cosmetic outcomes when performing BCS after NACT, especially for the patients with good response to NACT but whose residual tumor cells are scattered over the residual volume of disease. A consensus has been reached among the experts that “no ink on tumor” guideline was an adequate resection margin for BCS after NACT, because no relationship has been found between the margin width and outcomes (19, 20). Nowadays, the safety of BCS after NACT for the operable LABC remains controversial. Therefore, we conducted this study to compare the oncological outcomes after BCS versus mastectomy in patients receiving NACT.

## Materials and methods

### Study population

The data of patients treated with NACT between 2000 and 2014 in our institution were retrospectively reviewed. The inclusion criteria were 1) receiving NACT with large primary tumor and/or heavy axillary lymph nodal burden; 2) breast cancer with cT1-3N0-2M0 stage and ypT0-2N0-2M0 stage; 3) information available on estrogen receptor (ER), progesterone receptor (PR), and human epidermal growth factor receptor 2 (HER2) status; and 4) information available on whether or not to receive adjuvant treatment, such as chemotherapy, radiotherapy, endocrine therapy, or HER2-targeted therapy. The exclusion criteria were 1) received BCS without adjuvant radiotherapy; 2) mastectomy patients with proven ypN1-2 stage but no postmastectomy radiotherapy (PMRT); 3) relapse within 2 months; or 4) failure to complete at least 6 months of follow-up after surgery. We also excluded the patients with cT4/ypT3-4/ypN3 disease, because none of them had received BCS in our initial cohort. This study was approved and the need for informed consent was waived by Ethics Committee of National Cancer Center/National Clinical Research Center for Cancer/Cancer Hospital, Chinese Academy of Medical Sciences and Peking Union Medical College (approval number: 15-057/984), as this was a retrospective analysis of chart data.

### Outcomes

Locoregional recurrence (LRR) was defined as recurrence in the ipsilateral breast or chest wall, ipsilateral axilla, supra- or infra-clavicular lymph nodes, or internal mammary lymph nodes. Distant metastasis (DM) was defined as evidence of metastatic disease beyond the locoregional regions. Disease-free survival (DFS) was calculated from the date of definitive surgery to the date of LRR, DM, death, or the last follow up. Breast cancer–specific survival (BCSS) was calculated from the date of start of NACT to the date of death from BC or last follow-up. Overall survival (OS) was calculated from the date of start of NACT to the date of death or the last follow up.

### Statistical analysis

The chi-square test was used to compare patient characteristics between the BCS and mastectomy groups. Multivariate Cox regression models were used to assess the impact of surgery methods on recurrence and survival after adjusting for confounding factors including treatment era, age, clinical stage, NACT cycles, response to NACT, histological grade, lymphovascular invasion (LVI), molecular subtype/trastuzumab, ypStage (pathologic stage after NACT), hormone receptor (HR) status/endocrine therapy, and adjuvant chemotherapy. To reduce the effect of selection bias and potential confounding factors, the differences in baseline covariates between BCS and mastectomy groups were balanced by the inverse probability of treatment weighting (IPTW) method (21). The IPTW approach attempts to mimic a situation in which treatment is randomly allocated to individuals. The Kaplan–Meier method was used for analysis of recurrence and survival before and after IPTW; the log-rank test was used for comparisons between the groups. Statistical analysis was performed using the SPSS 22.0 (IBM Corp., Armonk, NY, USA) and R 4.1.2 (https://www.r-project.org/). Two-sided P <.05 was considered statistically significant.

## Results

### Baseline characteristics

**Figure 1:**
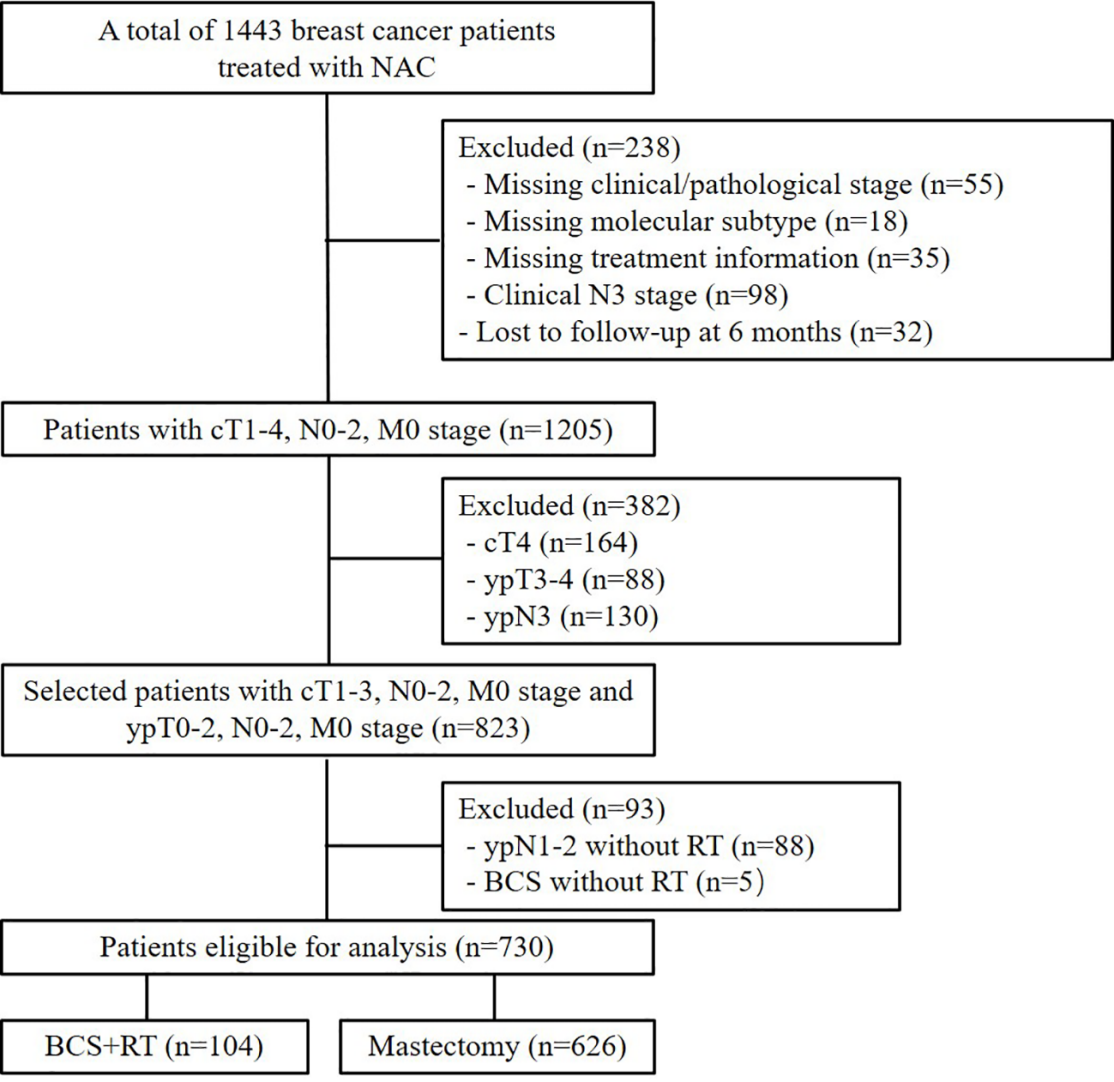
CONSORT diagram.

A total of 730 patients were enrolled in this analysis **(** **Figure 1** **).** **Table 1** summarizes the patients’ demographic characteristics and the tumor and treatment characteristics. The median age of the patients was 46 years (range, 20–73 years). Breast magnetic resonance imaging (MRI) was performed for 160 (21.9%) patients. Pretreatment median tumor diameter was 4 cm (range, 1–10 cm) in the BCS group and 5 cm (range, 1.1–13 cm) in the mastectomy group. There were 621 (85.1%), 80 (76.9%), and 541 (86.1%) patients who had tumor size ≥ 3cm in the entire, BCS, and mastectomy group, respectively. After NACT, there were 563 (77.1%), 97 (93.3%), and 466 (74.4%) patients who had pathologic tumor size < 3cm in the entire, BCS, and mastectomy group.

**Table 1:** Baseline characteristics of the study populations before and after IPTW (n = 730).

Variables	Unweighted population N (%)			Weighted population, N (%)		
	BCS 104 (14.2%)	Mastectomy 626 (85.8%)	*P*	BCS 837.5 (53.6%)	Mastectomy 725.1 (46.4%)	*P*
Treatment era						
**2000-2008**	38 (36.5)	223 (35.6)	.857	224.2 (26.8)	261.0 (36.0)	.240
**2009-2014**	66 (63.5)	403 (64.4)		613.2 (73.2)	464.0 (64.0)	
Age (y)						
**<50**	92 (88.5)	391 (62.5)	**<.001**	669.9 (80.0)	478.3 (66.0)	.091
**≥50**	12 (11.5)	235 (37.5)		167.6 (20.0)	246.7 (34.0)	
Clinical T stage						
**1**	6 (5.8)	28 (4.5)	.062	32.7 (3.9)	33.9 (4.7)	.892
**2**	70 (67.3)	354 (56.5)		511.2 (61.0)	419.9 (57.9)	
**3**	28 (26.9)	244 (39.0)		293.6 (35.1)	271.3 (37.4)	
Clinical N stage						
**0**	37 (35.6)	120 (19.2)	**<.001**	131.4 (15.7)	153.4 (21.2)	.572
**1**	62 (59.6)	405 (64.7)		534.2 (63.8)	465.4 (64.2)	
**2**	5 (4.8)	101 (16.1)		171.8 (20.5)	106.3 (14.6)	
Clinical stage						
**II**	77 (74.0)	360 (57.5)	**.001**	504.7 (60.3)	432.1 (59.6)	.948
**III**	27 (26.0)	266 (42.5)		332.7 (39.7)	293.0 (40.4)	
NACT cycles						
**≤4**	65 (62.5)	484 (77.3)	**.002**	669.7 (80.0)	548.2 (75.6)	.484
**>4**	39 (37.5)	142 (22.7)		167.7 (20.0)	176.8 (24.4)	
Response to NACT						
**CR**	9 (8.7)	79 (12.6)	.495	69.1 (8.3)	88.3 (12.2)	.059
**PR**	83 (79.8)	472 (75.4)		731.3 (87.3)	552.4 (76.2)	
**SD+PD**	12 (11.5)	75 (12.0)		37.0 (4.4)	84.4 (11.6)	
Histological grade						
**I+II**	59 (56.7)	303 (48.4)	.106	447.8 (55.4)	360.2 (49.7)	.446
**III**	17 (16.3)	161 (25.7)		222.6 (26.6)	178.7 (24.7)	
**Unknown**	28 (26.9)	162 (25.9)		133.3 (15.9)	186.2 (25.7)	
LVI						
**No**	96 (92.3)	564 (90.1)	.596	792.3 (94.6)	656.3 (90.5)	.307
**Yes**	8 (7.7)	62 (9.9)		45.2 (5.4)	68.8 (9.5)	
Molecular subtype/trastuzumab						
**Luminal (HER2−)**	57 (54.8)	281 (44.9)	**.013**	400.9 (47.9)	334.3 (46.1)	.564
**HER2+Trastu+**	15 (14.4)	78 (12.5)		83.7 (10.0)	91.2 (12.6)	
**HER2+Trastu−**	11 (10.6)	158 (25.2)		256.8 (30.7)	169.8 (23.4)	
**Triple negative**	21 (20.2)	109 (17.4)		96.1 (11.5)	129.8 (17.9)	
ypT stage						
**0-Tis**	22 (21.2)	132 (21.1)	**<.001**	122.3 (14.6)	153.4 (21.2)	.347
**1**	67 (64.4)	250 (39.9)		318.1 (38.0)	312.4 (43.1)	
**2**	15 (14.4)	244 (39.0)		397.0 (47.4)	259.3 (35.8)	
ypN stage						
**0**	60 (57.7)	312 (49.8)	.071	406.2 (48.5)	371.0 (51.2)	.964
**1**	30 (28.8)	167 (26.7)		241.6 (28.9)	193.6 (26.7)	
**2**	14 (13.5)	147 (23.5)		189.6 (22.6)	160.5 (22.1)	
ypStage						
**0**	20 (19.2)	106 (16.9)	**.006**	112.6 (13.4)	125.6 (17.3)	.834
**I**	33 (31.7)	117 (18.7)		150.6 (18.0)	149.4 (20.6)	
**II**	37 (35.6)	256 (40.9)		384.6 (45.9)	289.5 (39.9)	
**III**	14 (13.5)	147 (23.5)		189.6 (22.6)	160.5 (22.1)	
HR status/endocrine therapy						
**HR+ET+**	65 (62.5)	382 (61.0)	.288	626.9 (74.9)	445.1 (61.4)	.104
**HR+ET−**	8 (7.7)	28 (4.5)		53.0 (6.3)	34.3 (4.7)	
**HR−**	31 (29.8)	216 (34.5)		157.6 (18.8)	245.7 (33.9)	
Adjuvant chemotherapy						
**No**	39 (37.5)	132 (21.1)	**<.001**	152.2 (18.2)	166.5 (23.0)	.401
**Yes**	65 (62.5)	494 (78.9)		685.2 (81.8)	558.6 (77.0)	

IPTW, inverse probability of treatment weighting; BCS, Breast conserving surgery; NACT, neoadjuvant chemotherapy; PR, partial remission; CR, complete remission; SD, stable disease; PD, progressive disease; LVI, lymphovascular invasion; HER2, human epidermal growth factor receptor 2; Trastu+, with trastuzumab; Trastu-, without trastuzumab; ypT stage, pathologic tumor stage after NACT; ypN, pathologic lymph node stage after NACT; ypStage, pathologic stage after NACT; HR, hormone receptor; ET, endocrine therapy.

All 730 patients received NACT, with a median of 4 cycles (range, 1–8 cycles); 704 (96.4%) patients received anthracycline- and/or taxane-based regimens. After NACT, 104 (14.2%) patients received BCS and 626 (85.8%) received modified radical mastectomy. Seven (1.0%) patients underwent sentinel lymph node biopsy and 723 (99.0%) underwent axillary lymph node dissection; the median number of axillary lymph nodes removed was 19 (range, 1-44). After resection of the primary tumor, positive margin was found in 5 (0.7%) patients; all were focal and in the BCS group. After surgery, 126 (17.3%) patients achieved pathologic complete response (pCR), defined as breast pCR (ypT0 and ypTis) and axillary pCR (ypN0). Adjuvant chemotherapy was administered to 559 (76.6%) patients, with median of 3 cycles (range, 1–9 cycles). A total of 483 (66.2%) patients had ER/PR-positive disease; among them, 447 (92.5%) received endocrine therapy. Of the 262 (35.9%) HER2-positive patients, 93 (35.5%) received HER2-targeted therapy with trastuzumab, because trastuzumab was approved by the Chinsese Food and Drug Administration in September 2007.

In the BCS group, all 104 patients received whole-breast irradiation, 102 (98.1%) received tumor-bed boost, 42 (40.4%) received supra/infraclavicular nodal irradiation, and 1 (1.0%) received internal mammary nodal irradiation. Information on radiotherapy dose was available for 91 (87.5%) patients. The median dose delivered to the whole breast ± nodal regions was 50 Gy (range, 48–50 Gy) in 25 fractions (range, 24–25) for 86/91 (94.5%) patients or 43.5 Gy in 15 fractions for 5/91 (5.5%) patients. The median tumor-bed boost dose was 10 Gy (range, 10–20 Gy) in 5 fractions (range, 5–10) for 86/91 (94.5%) patients or 8.7 Gy in 3 fractions for 5/91 (5.5%) patients. Information on radiotherapy technique was available for 87 (83.7%) patients; while 29/87 (33.3%) received two-dimensional radiotherapy, 6/87 (6.9%) received three-dimensional conformal radiotherapy (3DCRT), and 52/87 (59.8%) received intensity-modulated radiotherapy (IMRT).

PMRT was recommended for patients with ypN1-2 stage and for ypN0 patients with high-risk factors (i.e., age < 45 years, cT3, cN2, presence of LVI, or ER/PR negative status). In the mastectomy group, 442 (70.6%) patients underwent PMRT. Information on radiotherapy fields was available for 415/442 (93.9%) patients. All 415 patients received chest wall irradiation, 407/415 (98.1%) received supra/infraclavicular nodal irradiation, 18/415 (4.3%) received axillary nodal irradiation, and 14/415 (3.4%) received internal mammary nodal irradiation. Information on radiotherapy dose was available for 379/442 (85.7%) patients; the median dose was 50 Gy (range, 42–60 Gy) in 25 fractions (range, 21–30) for 329/379 (86.8%) patients and 43.5 Gy in 15 fractions for 50/379 (13.2%) patients. Information on radiotherapy technique was available for 396/442 (89.6%) patients; while 381/396 (96.2%) received two-dimensional radiotherapy, 6/396 (1.5%) received 3DCRT, and 9/396 (2.3%) received IMRT.

Compared with mastectomy patients, BCS patients were significantly younger, had earlier clinical and pathological stage, and more likely to receive adjuvant chemotherapy. After IPTW adjustment, there were 837.5 (53.6%) patients in the BCS group and 725.1 (46.4%) patients in the mastectomy group, and clinical characteristics were comparable between the two groups **(** **Table 1** **)**.

### Treatment outcomes

The median follow-up of BCS and mastectomy groups were 86.5 months (range, 6.5-169.9 months) and 87.4 months (range, 6.1-201.3 months), respectively. A total of 58 (7.9%) patients had developed LRR, 159 patients (21.8%) had developed DM, and 83 (11.4%) patients had died. Among the 83 patients who died, 78 (94.0%) died of the BC and 5 (6.0%) died of other causes. All five patients that died from other causes were in the mastectomy group; the causes of death included pulmonary fibrosis (n = 1), acute pancreatitis (n = 1), leukemia (n = 1), anemia and thrombocytopenia (n = 1), and unknown cause (n = 1). The patient who died from acute pancreatitis had hypertension, gastric ulcer, and hyperthyroidism; the other four patients had no comorbidity at the time of diagnosis of BC.

**Figure 2:**
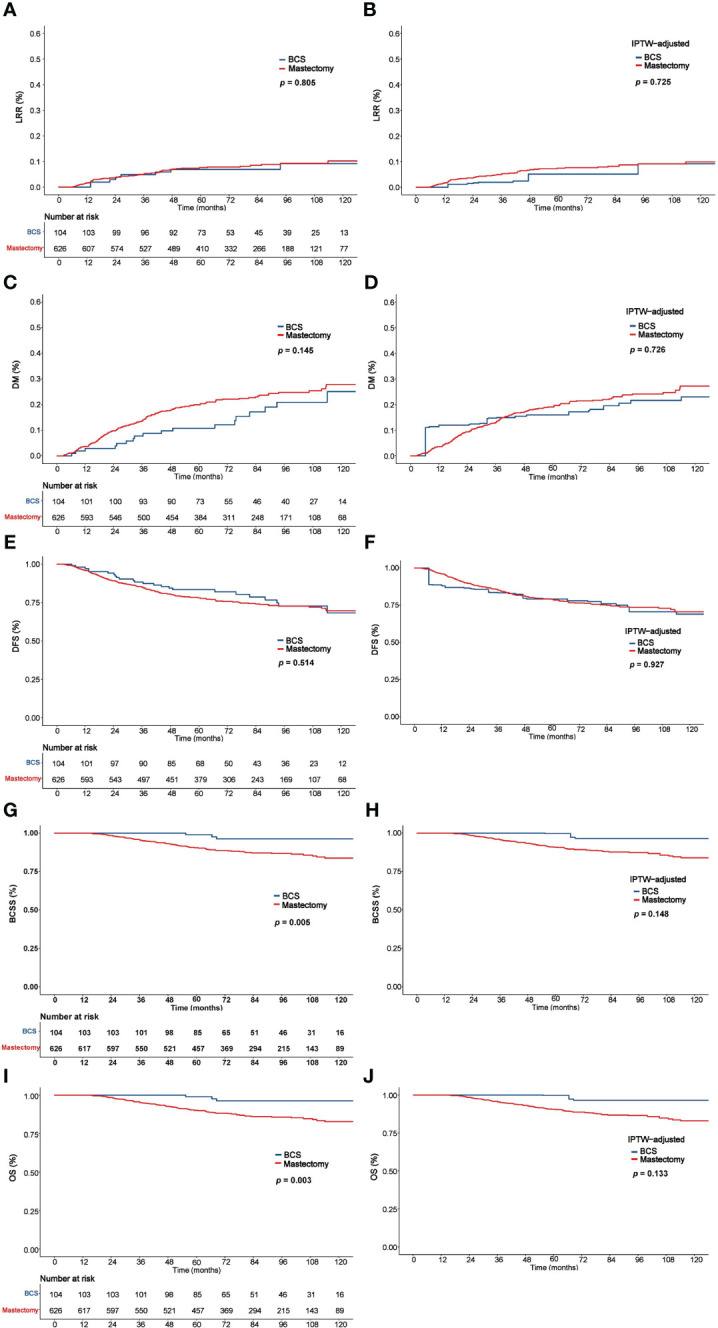
Comparison of LRR, DM, DFS, BCSS, and OS between the BCS and mastectomy groups before and after IPTW analysis. LRR **(A)**, DM **(C)**, DFS **(E)**, BCSS **(G)**, and OS **(I)** before IPTW analysis. LRR **(B)**, DM **(D)**, DFS **(F)**, BCSS **(H)**, and OS **(J)** after IPTW analysis. LRR, Locoregional recurrence; DM, distant metastasis; DFS, disease-free survival; BCSS, Breast cancer–specific survival; OS, overall survival; IPTW, inverse probability of treatment weighting.

The 5-year LRR, DM, DFS, BCSS, and OS rates in the entire cohort were 7.5%, 18.6%, 78.9%, 91.7%, and 91.4%, respectively. As **Figure 2** shows, there were no significant differences between the mastectomy group and the BCS group in 5-year LRR (6.9% vs. 7.6%, P = 0.805), DM (10.8% vs. 19.9%, P = 0.145), and DFS (83.4% vs. 78.2%, P = 0.514); however, the BCS group had significantly better BCSS (98.9% vs. 90.4%, P = 0.005) and OS (98.9% vs. 90.1%, P = 0.003).

Multivariate analysis did not reveal significant differences between the BCS and mastectomy groups in LRR (HR = 1.15, 95% CI: 0.52-2.56, P = 0.731), DM (HR = 0.80, 95% CI: 0.48-1.34, P = 0.400), and DFS (HR = 1.06, 95% CI: 0.67-1.68, P = 0.809); however, the BCS group had significantly better BCSS (HR = 0.27, 95% CI: 0.08-0.85, P = 0.025) and OS (HR = 0.25, 95% CI: 0.08-0.79, P = 0.018) **(** **Table 2** **)**. Further, multivariate analysis showed clinical stage, ypStage, and HR status to be independent predictors of prognosis.

**Table 2:** Multivariate analysis of the entire cohort.

Characteristics	LRR			DM			DFS			BCSS			OS		
	HR	95% CI	*P*	HR	95% CI	*P*	HR	95% CI	*P*	HR	95% CI	*P*	HR	95% CI	*P*
**Treatment era (2009-2014 vs. 2000-2008)**	0.603	0.356-1.020	.059	0.840	0.592-1.190	.326	0.746	0.548-1.016	.063	0.922	0.558-1.523	.752	0.901	0.557-1.459	.673
**Age (≥50 vs. <50)**	0.934	0.534-1.635	.812	1.006	0.721-1.402	.973	1.069	0.782-1.461	.676	1.107	0.700-1.751	.665	1.102	0.707-1.719	.668
**Clinical stage (III vs. II)**	1.559	0.925-2.625	.095	1.350	0.969-1.880	.076	1.434	1.066-1.928	.017	1.216	0.758-1.950	.417	1.280	0.809-2.027	.292
**NACT cycles (>4 vs. ≤4)**	0.927	0.333-2.583	.885	1.261	0.698-2.276	.443	1.054	0.601-1.848	.855	1.219	0.514-2.891	.653	1.171	0.500-2.742	.715
**Response to NACT**			.663			.525			.708			.863			.910
**PR vs. CR**	1.886	0.472-7.534	.369	0.920	0.418-2.026	.837	1.311	.603-2.849	.495	1.032	.327-3.252	.958	1.131	0.364-3.511	.832
**SD+PD vs. CR**	1.764	0.372-8.363	.475	1.187	0.492-2.862	.703	1.441	0.607-3.424	.408	1.228	.342-4.406	.753	1.272	0.360-4.499	.709
**Histological grade**			.760			.288			.476			.430			.438
**III vs. I+II**	0.879	0.451-1.715	.705	0.737	0.483-1.124	.156	0.789	0.532-1.169	.237	0.829	0.469-1.463	.517	0.923	0.537-1.584	.770
**Unknown vs. I+II**	0.777	0.393-1.534	.467	0.798	0.531-1.199	.277	0.881	0.605-1.282	.507	0.672	0.364-1.240	.204	0.678	0.374-1.230	.201
**LVI (Yes vs. No)**	1.515	0.745-3.082	.252	0.861	0.520-1.423	.559	1.012	0.640-1.601	.960	0.822	0.389-1.740	.609	0.776	0.368-1.636	.505
**Molecular subtype/trastuzumab**			.405			.863			.890			.717			.806
**HER2+/Trastu+ vs. Luminal (HER2-)**	2.057	0.846-5.002	.112	1.083	0.604-1.944	.788	1.237	0.721-2.123	.440	0.969	0.401-2.342	.944	0.952	0.398-2.277	.912
**HER2+/Trastu- vs. Luminal (HER2-)**	1.083	0.489-2.397	.844	0.863	0.535-1.393	.546	1.082	0.697-1.682	.724	0.710	0.340-1.485	.363	0.831	0.411-1.680	.606
**Triple negative vs. Luminal (HER2-)**	1.611	0.522-4.978	.407	1.013	0.496-2.069	.971	1.083	0.563-2.080	.812	0.649	0.242-1.746	.392	0.655	0.253-1.691	.382
**ypStage**			<.001			<.001			<.001			<.001			<.001
**I vs. 0**	0.850	0.245-2.943	.797	1.640	0.731-3.680	.230	1.379	0.669-2.846	.384	5.905	1.280-27.239	.023	6.762	1.484-30.805	.013
**II vs. 0**	1.931	0.733-5.082	.183	3.661	1.820-7.366	<.001	3.041	1.650-5.606	<.001	8.067	1.922-33.854	.004	9.051	2.166-37.820	.003
**III vs. 0**	3.963	1.514-10.378	.005	7.701	3.795-15.627	<.001	5.851	3.135-10.918	<.001	19.112	4.543-80.400	<.001	20.433	4.867-85.780	<.001
**Surgery (BCS vs. Mastectomy)**	1.150	0.517-2.558	.731	0.801	0.477-1.344	.400	1.058	0.668-1.676	.809	0.265	0.083-0.845	.025	0.249	0.078-0.791	.018
**HR status/endocrine therapy**			.908			.654			.653			.008			<.001
**HR+/ET- vs. HR+/ET+**	0.773	0.182-3.281	.727	1.243	0.594-2.598	.564	1.061	0.510-2.208	.873	1.551	0.555-4.338	.403	1.526	0.546-4.262	.420
**HR- vs. HR+/ET+**	1.111	0.466-2.651	.813	1.251	0.714-2.191	.434	1.266	0.767-2.090	.357	2.093	1.313-3.334	.002	2.288	1.460-3.584	<.001
**Adjuvant chemotherapy (No vs. Yes)**	1.148	0.398-3.313	.798	1.412	0.958-2.082	.082	1.394	0.955-2.036	.086	1.018	0.412-2.513	.969	0.972	0.398-2.372	.951

LRR, Locoregional recurrence; DM, distant metastasis; DFS, disease-free survival; BCSS, Breast cancer–specific survival; OS, overall survival; HR, hazard ratio; CI, confidence interval; NACT, neoadjuvant chemotherapy; PR, partial remission; CR, complete remission; SD, stable disease; PD, progressive disease; LVI, lymphovascular invasion; HER2, human epidermal growth factor receptor 2; Trastu+, with trastuzumab; Trastu−, without trastuzumab; ypStage, pathologic stage after NACT; BCS, Breast conserving surgery; HR, hormone receptor; ET, endocrine therapy.

In the IPTW-adjusted cohort, 5-year LRR, DM, DFS, BCSS and OS were comparable between the BCS and mastectomy groups, which were 5.1% vs. 7.4% (P = 0.725), 16.0% vs. 19.1% (P = 0.726), 79.1% vs. 79.0% (P = 0.927), 99.7% vs. 90.8% (P = 0.148), and 99.7% vs. 90.3% (P = 0.133), respectively **(** **Figure 2** **)**.

## Discussion

In this single-center cohort study, we retrospectively evaluated the oncologic safety of BCS compared with mastectomy following NACT in patients with BC and found that LRR, DM, DFS, BCSS, and OS are comparable with BCS and mastectomy. Overall, our findings suggest that BCS is a safe and effective treatment option after NACT for patients with BC.

In recent years, NACT has become standard treatment for unresectable or resectable LABC and is being increasingly used in early-stage BC. The NSABP B18 trial examining the sequencing of chemotherapy demonstrated that patients treated with NACT had similar survival outcomes with those treated with adjuvant chemotherapy and NACT could increase BCS rates by tumor down-staging (11). The ipsilateral breast tumor recurrence (IBTR) rates were comparable in the NACT and adjuvant chemotherapy groups (7.9% vs. 5.8%). However, among women being converted from mastectomy to BCS candidates after NACT, the IBTR rate was higher (14.3%). Thus, the effectiveness of BCS after NACT remains unclear. Chen et al. reported acceptably low 5-year IBTR and LRR rates of 5% and 9% for the patients treated with BCS after NACT in a cohort of 340 patients from the MD Anderson Cancer Center; however, women with any one of the high-risk factors (i.e., cN2-3, pathologically residual tumor > 2 cm, multifocal residual disease, and LVI) had higher IBTR and LRR rates (9%–13% and 16%–23%) (18).

In our study, the surgical method was selected based on tumor response to NACT and patients’ preference. Given the small breast size for most eastern women and few applications of oncoplastic surgery, the guidelines recommended that BCS should be offered to the patients who had tumor size < 3cm or had appropriate ratio of tumor to breast volume to achieve good cosmesis. In our study, 621 (85.1%) patients had tumor size ≥ 3cm who were not good candidates for BCS initially. After NACT, 563 (77.1%) had pathologic tumor size < 3cm who were presumed to be appropriate for BCS. However, most patients selected to receive mastectomy, which constituted a comparable cohort for the present study. In the present study, the LRR, DM, and DFS were similar in BCS and mastectomy patients; BCSS and OS were better in BCS patients, although the differences were not statistically significant after confounding factors were adjusted by IPTW. A meta-analysis found no significant difference in local and regional recurrence between BCS and mastectomy patients after NACT and that BCS patients had lower incidence of DM and better DFS and OS (22). Previous studies that reported the oncologic safety of BCS after NACT are summarized in **Table 3** (13, 23–36). All studies were retrospective and most had small sample size. In a cohort of 561 patients treated with NACT, Simons et al. found significantly better DFS and OS after BCS than after mastectomy, but the statistical significance disappeared after correcting for confounders (36). An analysis of population-based data from the New Jersey State Cancer Registry (NJSCR) demonstrated that BCS patients after NACT had significantly better 10-year BCSS than mastectomy patients, and the difference remained even in propensity-matched comparison. However, the study findings must be interpreted cautiously because all patients did not receive radiotherapy after mastectomy and some important clinical factors such as tumor size, radiotherapy and chemotherapy data were missing in the database (35). Barranger et al. reported a 72.3% “mastectomy to BCS” conversion rate after NACT for resectable LABC. For whom mastectomy was the only conceivable surgical option initially, the 5-year DFS and OS of BCS and mastectomy patients were comparable (74% vs. 59% and 77% vs. 77%, respectively). But baseline tumor characteristics were not balanced between the two groups, the BCS patients having smaller tumor size and higher pCR rate, which were not adjusted during survival analysis and would have affected the outcomes (13). Most of the aforementioned studies reported results that were consistent with ours, i.e., that BCS after NACT is a safe alternative to mastectomy in patients with BC; however, these earlier studies had obvious limitations such as unbalanced baseline characteristics and missing important data. In our study, we included patients who had received adjuvant radiotherapy per the current guideline (e.g., radiotherapy after BCS, PMRT if ypN1-2 or ypN0 with high-risk factors). Confounding due to differences in baseline covariates between the two surgical groups was minimized by using the IPTW method. These measures make our results more convincing. All five patients who died from non-breast-cancer causes were in the mastectomy group, but most had no comorbidities at diagnosis of BC; therefore, the poorer OS in the mastectomy group might not be related to presence of comorbidities.

**Table 3:** Summary of previous studies comparing BCS and mastectomy after NACT in patients with breast cancer.

Author, Year	Enrollment Period	Median follow-up, mo	Eligibility (No.)	Surgical method	Response to NACT			Median tumor size (cm)		LRR(%)	DM(%)	5-year DFS(%)	5-year OS(%)
					CR (%)	PR (%)	SD+PD (%)	Pre-NACT	Post-NACT				
Schwartz, 1994 (23)	1983-1991	46	T2N2, T3-4N0-2 (n=158)	BCS	100		–	NA	NA	NA	NA	77	80
				Mastectomy				NA	NA	NA	NA	56	67
Cance, 2002 (24)	1992-1998	70	T3-4, N2 (n=59)	BCS	22	76	2	NA	NA	10	NA	NA	96*
				Mastectomy				NA	NA	16	NA	NA	51*
McIntosh, 2003 (25)	1992-1997	62	T2 > 4cm, T3-4N0-1 (n=166)	BCS	21	54	25	NA	1.3	2.3	NA	NA	MST: 75m
				Mastectomy				NA	3.4	5.2	NA	NA	MST: 22m
Rouzier, 2004 (26)	1987-2001	67	T2-3N0-2 (n=594)	BCS	14.6	59.6	25.8	4.9	3.1	NA	25*	NA	NA
				Mastectomy	2.9	35.2	61.9			NA	37*	NA	NA
Sadetzki, 2005 (27)	1995-2001	>27	Stage II > 3cm, III (n=119)	BCS	NA	NA	NA	4.67	1.68	NA	NA	NA	NA
				Mastectomy	NA	NA	NA	4.74	3.29	NA	NA	NA	NA
Parmar, 2006 (28)	1998-2002	30	LABC (n=664)	BCS	92.7		7.3	6	1.5	NA	11.1*	62*	NA
				Mastectomy	67.1		32.9	8.3	4.1	NA	25.6*	37*	NA
Sweeting, 2011 (29)	1991-2007	76.8	Stage II, III (n=122)	BCS	43	44	13	5.6	1.3	13	NA	82*	88*
				Mastectomy				6.7	3.2	18	NA	58*	61*
Cho, 2013 (30)	1998-2010	45.9	pathologic tumor size ≤3 cm (n=431)	BCS	30.6	NA	NA	NA	NA	5.5	11.0	80.7	89.1
				Mastectomy	11.1	NA	NA	NA	NA	6.2	16.0	74.6	84.2
Shin, 2013 (31)	2004-2007	62.4	Stage III (n=129)	Preplanned BCS	NA	NA	NA	NA	< 4	5.3 (LR)	NA	NA	NA
				Downstaged BCS	NA	NA	NA	NA		9.1 (LR)	NA	NA	NA
				Mastectomy	NA	NA	NA	NA		3.7 (LR)	NA	NA	NA
Levy, 2014 (32)	2002-2012	75.6	Stage I-III (n=284)	BCS	27	NA	NA	4	NA	9.2 (10y)	27 (10y)	NA	63 (10y)
				Mastectomy	5	NA	NA	5	NA	10.7 (10y)	41 (10y)	NA	60 (10y)
Cureton, 2014 (33)	2002-2006	46.8	Clinical tumor size ≤3 cm (n=206)	BCS	NA	NA	NA	6	NA	NA	16.9	NA	NA
				Mastectomy	NA	NA	NA		NA	NA	23.9	NA	NA
Barranger, 2015 (13)	2007-2012	41.1	Candidates for Mastectomy initially (n=119)	BCS	NA	NA	NA	3.4	1.7	3.5	NA	74	77
				Mastectomy	NA	NA	NA	5.5	3.3	3.0	NA	59	77
Debled, 2015 (34)	2005-2012	38	cT2-4, HER2+ (n=152)	BCS	NA	NA	NA	4.5	NA	5.6 (LR)	15.7	NA	NA
				Mastectomy	NA	NA	NA	7.0	NA	0	25	NA	NA
Arlow, 2018 (35)	1998-2003	110.5	BCS with RT, Mastectomy without RT (n=718)	BCS	NA	NA	NA	NA	NA	NA	NA	NA	BCS had better BCSS
		106.0		Mastectomy	NA	NA	NA	NA	NA	NA	NA	NA	
Simons, 2020 (36)	2008-2017	81.6	cT1-4N0-N+M0 (n=561)	BCS	25.7	NA	NA	NA	NA	NA	NA	90.9*	95.3*
				Mastectomy	19.1	NA	NA	NA	NA	NA	NA	82.9*	85.9*
Our study	2000-2014	87.4	cT1-3N0-2M0 and ypT0-2N0-2M0 (n=730)	BCS	8.7	79.8	11.5	4.0	1.0	6.9 (5y)	10.8 (5y)	83.4	98.9*
				Mastectomy	12.6	75.4	12.0	5.0	2.0	7.6 (5y)	19.9 (5y)	78.2	90.1*

* Statistically significant.

BCS, Breast conserving surgery; NACT, neoadjuvant chemotherapy; CR, complete remission; PR, partial remission; SD, stable disease; PD, progressive disease; LRR, Locoregional recurrence; DM, distant metastasis; DFS, disease-free survival; OS, overall survival; NA, not available; MST, median survival time; LABC, locally-advanced breast cancer; LR, Local recurrence; HER2, human epidermal growth factor receptor 2; RT, radiotherapy; BCSS, breast cancer–specific survival.

In multivariate analysis, ypStage was independent prognostic factors for most oncologic outcomes, and hormone receptor–negative status was a significant predictor of poor OS. As other authors have also demonstrated that advanced post-NACT stage and triple-negative status were significant predictors of poor outcome; this is not surprising since these factors indicate aggressive disease (30, 37).

Though this study suggests that BCS is a safe treatment option for patients after NACT, some concerns remain. In clinical practice, the primary tumor site is usually difficult to locate after tumor regression. One study that assessed pathological response of BC to NACT found increased incidence of multifocality and *in situ* lesions localized within the original tumor-bearing area after tumor shrinkage (38); this can lead to difficulty in defining the extent of resection necessary to achieve safe margin during BCS. However, over the past few years, there have been major advances to improve the probability of safe BCS after NACT. These advances include increased application of breast MRI, use of metal markers to improve definition of tumor location, and improved detection of multifocal or multicentric tumor, greater attention to achieving pathologically negative BCS margins and use of modern radiotherapy techniques that provide more precise dose coverage and thus improve local control and decrease toxicity. A policy review endorsed by several European societies and clinical trial groups has provided a practical working toolbox for the surgical treatment of early-stage BC after NACT (39). There is now consensus that all patients receiving NACT must undergo comprehensive evaluation in multidisciplinary team meetings, undergo imaging by multiple modalities (e.g., MRI and ultrasound) at diagnosis, and have clips placed at the primary site before NACT. In addition, response assessment at different time points must be done by the same imaging modality used at initial diagnosis; careful preoperative evaluation for localization, volume excision, and retrieval of breast markers is essential before BCS. Precise margin assessment and appropriate radiotherapy are also important for successful BCS. Through the close cooperation of multidisciplinary team, not only do the patients with resectable LABC have an opportunity to be converted from mastectomy to BCS candidates after NACT, but the women with early-stage triple-negative or HER2 - positive BC who are currently candidates for NACT are safe to receive BCS.

Some limitation of this study should be acknowledged. First, the retrospective design might have introduced a selection bias. Although IPTW was used to balance known variables in the two groups, it is possible that other confounders were unevenly distributed. Second, the 15-year span of patient inclusion was very long; but the patients in different treatment era between the two groups were comparable, and the influences of the changes in the diagnosis and treatment of BC over this period between the two groups were similar, e.g. the proportion of the patients who had HER2 - positive disease but did not received trastuzumab-targeted therapy was comparable after IPTW between the two groups. Third, the findings of this study can only be applied to specific populations, i.e. patients with cT1-3N0-2M0 and ypT0-2N0-2M0 BC, and the effect of BCS in patients with more advanced stage remains to be accessed. To our knowledge, this is the largest study to compare the outcomes between BCS and mastectomy after NACT, thus, we believe that our study makes a meaningful contribution to clinical practice.

## Conclusions

Breast-conserving surgery appears to be a safe treatment option for selected BC patients after neoadjuvant chemotherapy. It does not compromise locoregional, distant control, DFS, BCSS and OS compared with mastectomy.

## Data availability statement

The raw data supporting the conclusions of this article will be made available by the authors, without undue reservation.

## Ethics statement

The studies involving human participants were reviewed and approved by Ethics Committee of National Cancer Center/National Clinical Research Center for Cancer/Cancer Hospital, Chinese Academy of Medical Sciences and Peking Union Medical College (approval number: 15-057/984). Written informed consent for participation was not required for this study in accordance with the national legislation and the institutional requirements.

## Author contributions

Y-CS: Formal analysis, investigation, data collection, methodology, and writing of the first draft. ZH: statistical analysis, investigation, data collection, methodology. HF, YuT, HJ, Y-WS, JJ, Y-PL, BC, YuanT, S-NQ, N-NL, and NL: Patient care and review, and editing of the manuscript. Y-XL and S-LW: Study design, formal analysis, validation, statistical analysis guidance, patient care, and editing of the manuscript. All authors contributed to the article and approved the submitted version.
